# Supplementary figures and images for: Post-insemination selection dominates pre-insemination selection in driving rapid evolution of male competitive ability
Source: PLoS Genet. 2022 Feb 14;18(2):e1010063. doi: 10.1371/journal.pgen.1010063 (PMC8880957; doi:10.1371/journal.pgen.1010063)

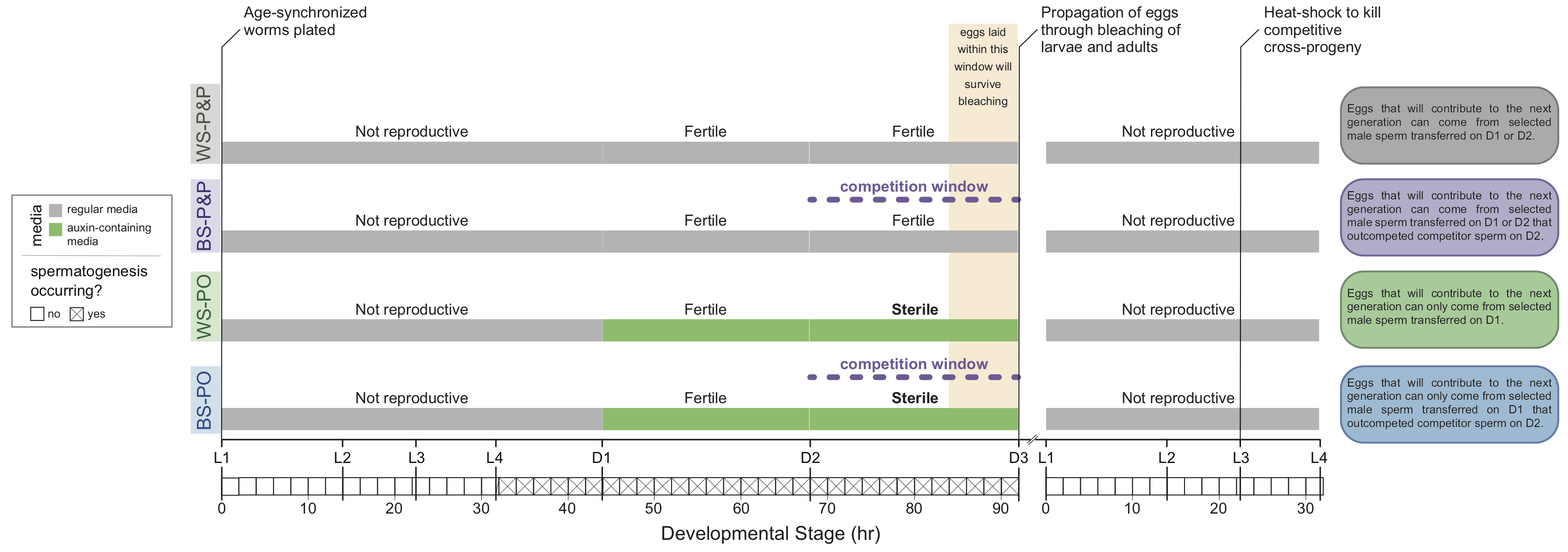

Supplement: S1 Fig — A selective event was initiated by plating age-synchronized larval stage 1 (L1) worms on regular nematode-growth media (shown in gray). Throughout larval development (L1-L4) worms were not reproductively active. However, spermatogenesis began in L4 males (shown by the crossed-out boxes) and therefore sperm was produced and stored prior to mating. Day 1 (D1) of adulthood corresponds to day 1 of the experimental timeline (see Fig 1). The WS-PO and BS-PO regimes were transferred to auxin-containing media (shown in green), while the WS-P&P and BS-P&P regimes remained on regular media. All males, regardless of regime, were fully fertile at the start of day 1. Females began laying eggs on day 1. On day 2 of adulthood, males on auxin-containing media (i.e., WS-PO and BS-PO) were fully sterile and could no longer transfer sperm. Competitor males were added to the BS-P&P and BS-PO regimes on day 2. Females laid eggs throughout adulthood, however, only eggs laid in the last 6–8 hours of day 2 had a thick enough eggshell to survive the propagation process. On day 3, populations were propagated by bleach-killing larvae and adults and then age-synchronizing surviving eggs. The resulting L1s were again plated on regular media and grown to L3 before heat-shocking to kill progeny coming from the competitor males. (TIFF) [file pgen.1010063.s001.tiff]

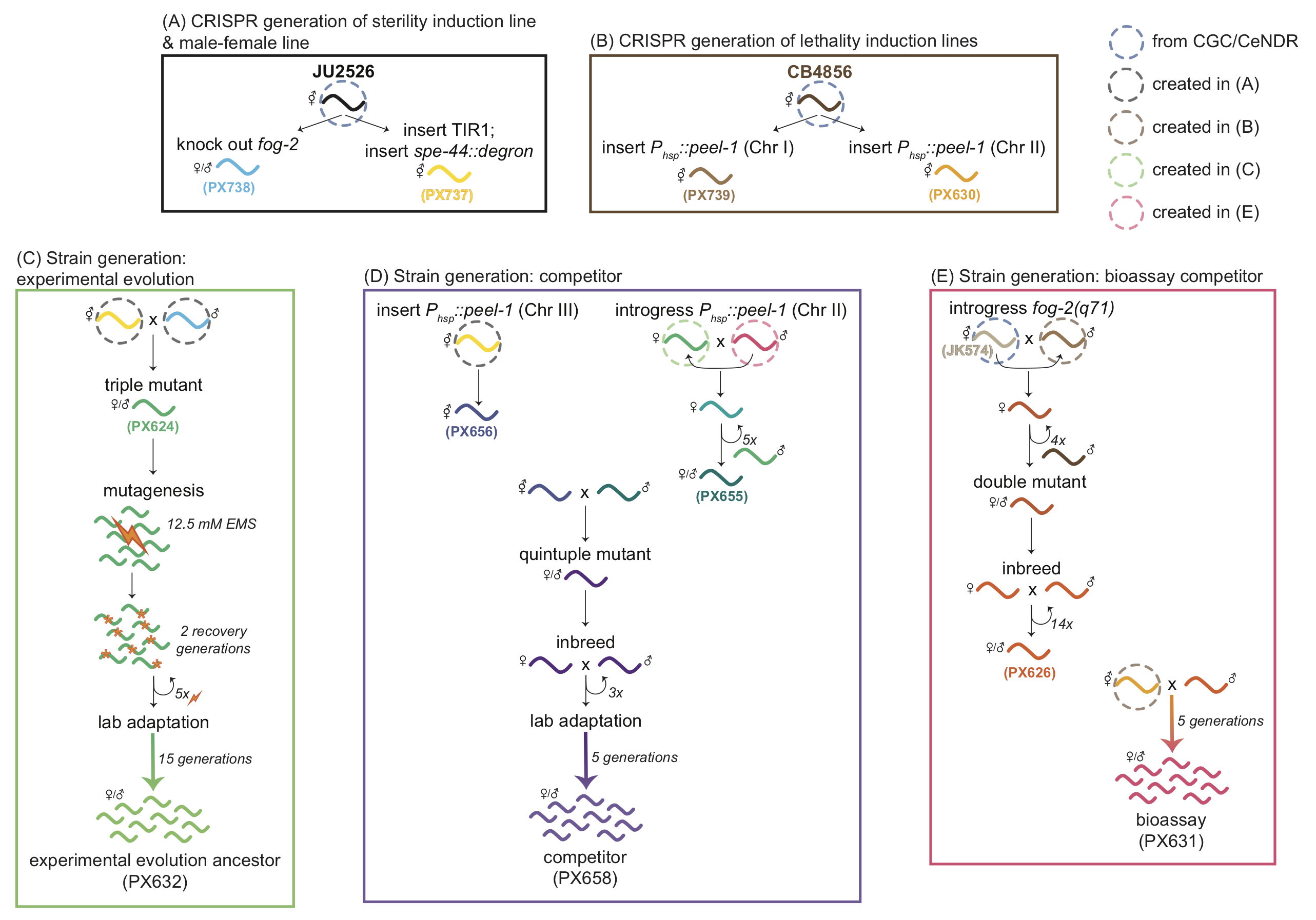

Supplement: S2 Fig — A) The components for creating an obligate outcrossing sterility induction line were genetically engineered in the wild isolate background JU2526. The spermatogenesis gene spe-44 was degron-tagged and TIR1 was inserted to create strain PX737. The hermaphrodite self-sperm gene (fog-2) was knocked-out to create strain PX738. These strains are used in panels C and D. B) To generate an inducible lethality line, heat-shock driven peel-1 was inserted into the CB4856 background on Chromosomes I and II to create strains PX739 and PX630, respectively. These strains are used in panels D and E. C) Strains PX737 and PX738 were crossed to creating a male-female, inducible sterility triple mutant (PX624). Strain PX624 went through five low dose rounds of mutagenesis each followed by two recovery generations. After the final recovery generation, the population was expanded for 15 generations of lab adaptation to create the experimental evolution ancestral population (PX632). D) The competition strain has five transgenic modifications. Heat-shock driven peel-1 was inserted on Chromosome III of strain PX737, creating an inducible lethality and inducible sterility strain (PX656). Strains PX624 and PX631 (panel E) were crossed to given another inducible lethality and sterility double mutant. These worms were backcrossed to PX624 five times to give a predominantly JU2526 genomic background. This strain, PX655, was crossed with PX656 yielding a quintuple mutant, which was inbred to three generations followed by five generations of lab adaptation. The final strain PX658 served as the competitor during experimental evolution. E) A separate bioassay competitor strain was generated by introgressing the fog-2(q71) mutation into PX739. These worms were backcrossed to the CB4856 genomic background four times and then inbred for 14 generations, creating strain PX626. This strain was crossed to PX630 to create an obligate outcrossing strain with two heat-shock driven peel-1 insertions. The final [file pgen.1010063.s002.tiff]

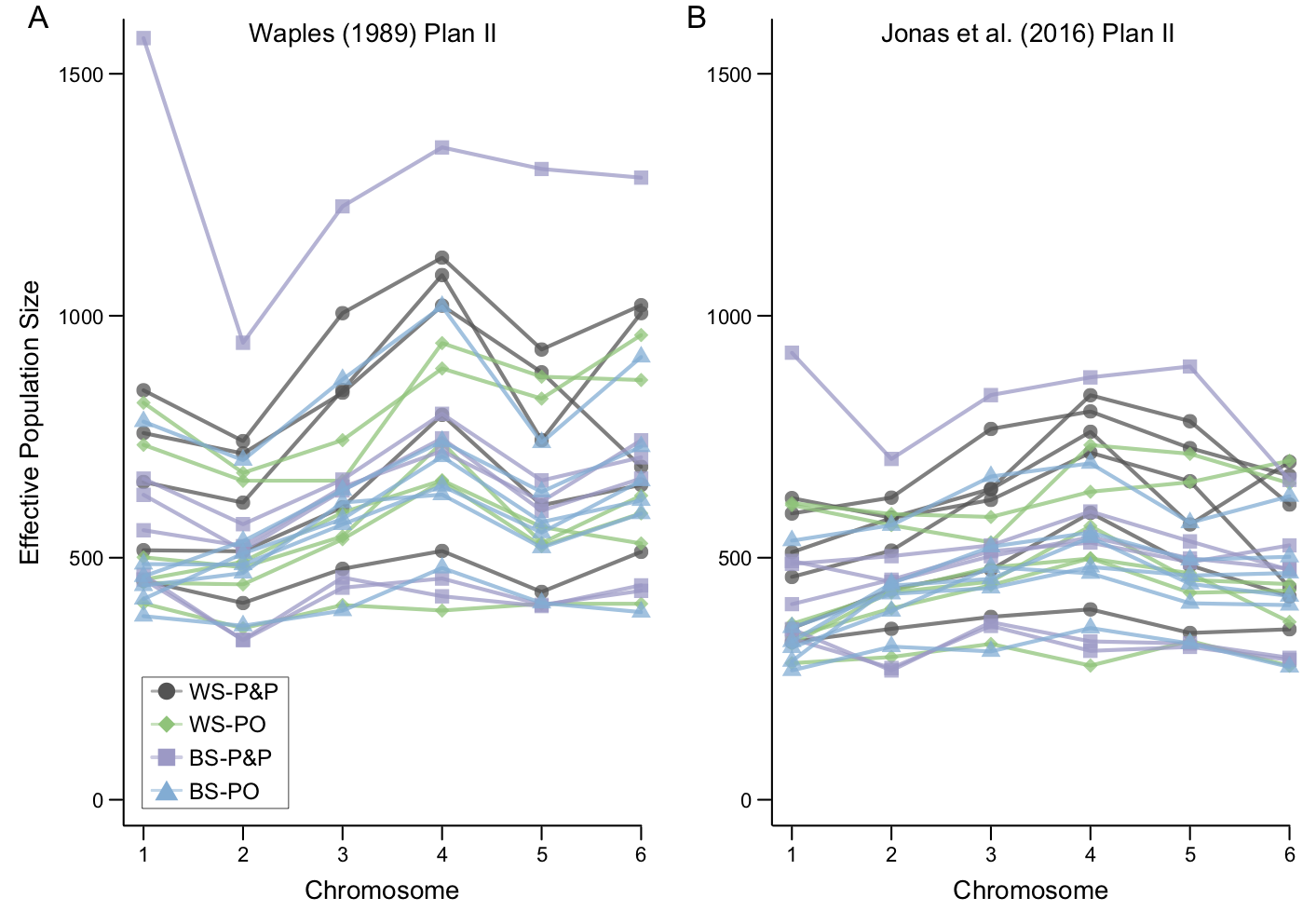

Supplement: S4 Fig — The effective population size was greatly reduced compared to the census size (N = 5,000). Regime did not have a significant effect on effective population size (Waples Plan II Sampling [60]: F = 0.98, DF = 3, p = 0.42; Jonas Plan II Sampling [61]: F = 0.79, DF = 3, p = 0.51). (TIFF) [file pgen.1010063.s004.tiff]

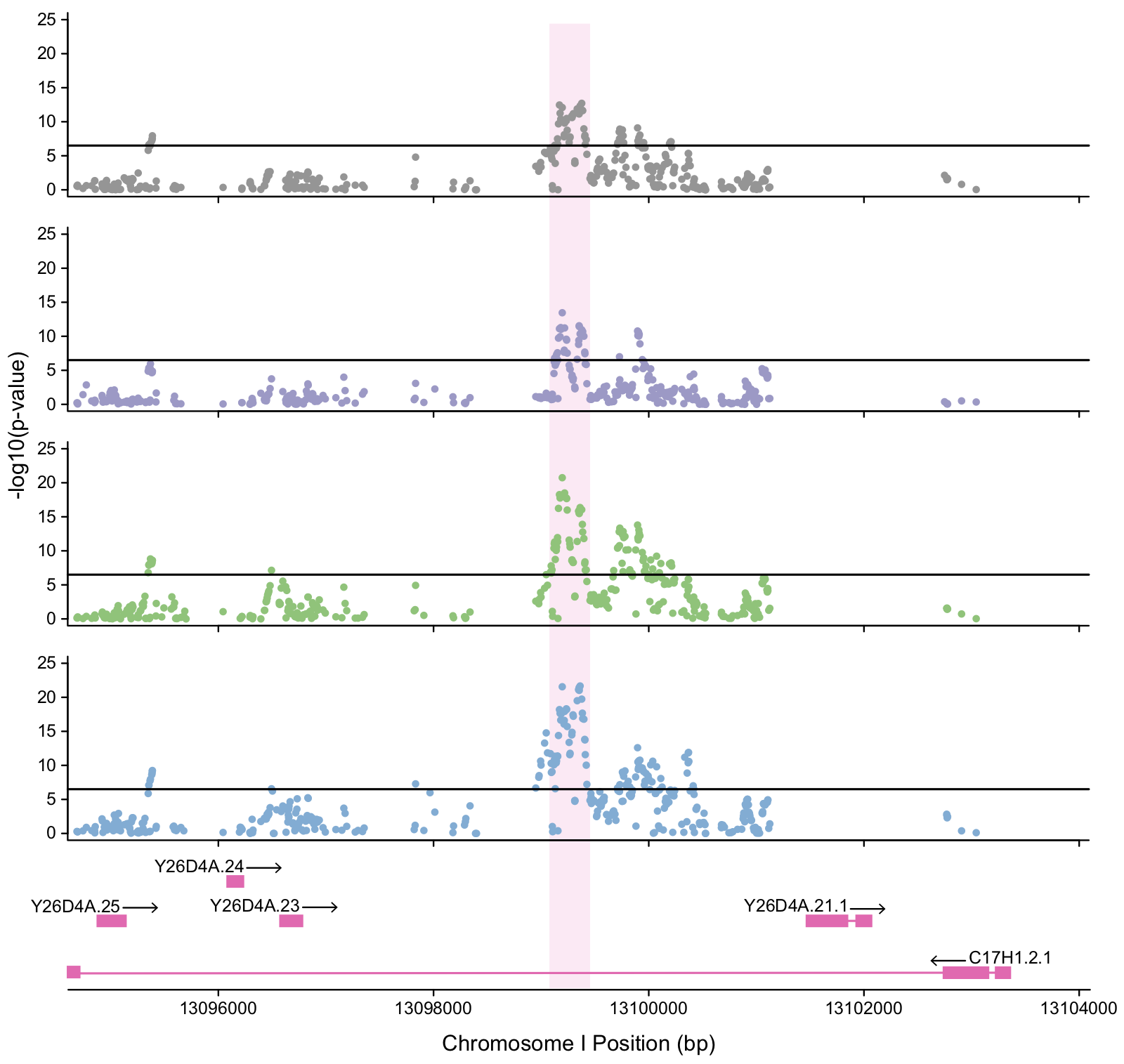

Supplement: S5 Fig — Significant SNPs pile up in the second intron of gene C17H1.2. This gene has male-biased expression, though it’s function is uncharacterized. (TIFF) [file pgen.1010063.s005.tiff]

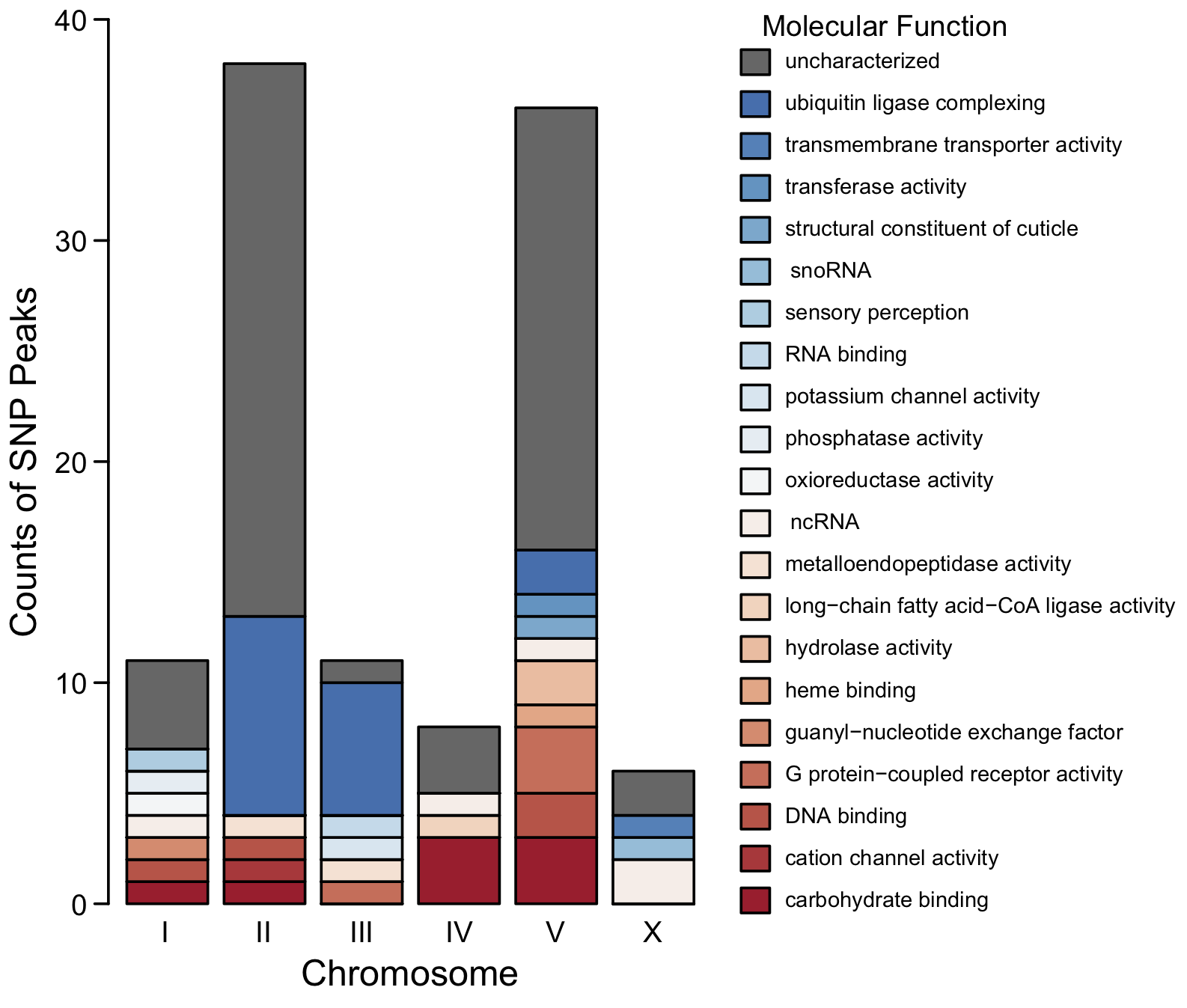

Supplement: S6 Fig — Ubiquitin ligase complex formation through F-box proteins, carbohydrate binding, G-coupled protein receptor activity, and DNA binding were the most common functions identified. However, the majority of genes are yet uncharacterized in function. (TIFF) [file pgen.1010063.s006.tiff]
