# Supplementary material for: Post-insemination selection dominates pre-insemination selection in driving rapid evolution of male competitive ability
Source: PLoS Genet. 2022 Feb 14;18(2):e1010063. doi: 10.1371/journal.pgen.1010063 (PMC8880957; doi:10.1371/journal.pgen.1010063)
Supplement: Attachment — Submitted filename: Response_to_Reviewers.pdf [file pgen.1010063.s020.pdf]

The manuscript was fully evaluated at the editorial level and by independent peer reviewers. The reviewers appreciated the attention to an important problem, but raised some substantial concerns about the current manuscript. Based on the reviews, we will not be able to accept this version of the manuscript, but we would be willing to review a revised version. We cannot, of course, promise publication at that time. In the revision, please ensure that the manuscript is framed for and accessible to a broad genetics audience. In addition, there are several questions about the experimental evolution protocol that need to be addressed, some of which include acknowledging the limitations of the design. Finally, the reviewers have many helpful suggestions about the Ne and pool-seq analyses that can make these analyses more robust.

> We thank the reviewers and editors for their thorough critique of our manuscript. We have revised our manuscript to incorporate all of the feedback. Of note, we have substantially expanded our explanation of the experimental design and added a new supplemental figure (Fig S1) that overlays the experiment details with *C. elegans* developmental time points. We have also re-calculated Ne and corrected the technical errors in our pool-seq analyses. We believe the revised version of the manuscript is improved and ready for publication in PLOS Genetics.

Reviewer #1

This is a really nice paper reporting interesting results from a very elegantly designed experiment. To my knowledge, this is the first careful dissection/quantitation of pre- and post-insemination selection and their relative efficacy in modulating overall reproductive success. Thus, the results are of non-trivial interest to those working on sexual selection and sexual conflict. Moreover, there is a lot of genomic information that points to future work in identifying important genes in the response to pre- and post-insemination selection. I have no substantive critiques; a few small comments/queries to authors are noted on the uploaded manuscript as pop-up notes.

> We appreciate the supportive feedback and address the minor comments from the manuscript pop-up notes below.

1. The balance is not needed for assessing male reproductive fitness. For that offspring rate will suffice.
   > Removed.

2. Is there any pattern to these mixed results in terms of what kinds of species show a greater role of variance in mating success? Might be worth saying a bit about any such pattern, or lack thereof.
   > We agree this is an interesting question. There appears to be no pattern across taxa based on the Evans and Garcia-Gonzalez review and a meta-analysis by Mautz et al. (reference added to introduction). The lack of pattern is likely in part due to the inconsistency in methods used across taxa, as noted in the introduction.

3. Not immediately clear how? A bit more explanation would be helpful.
   > We thank the reviewer for highlighting areas of the design that need clarification. We have expanded first section of the results and added Figure S1, which clarify the function of the sterility induction system.

4. It might be worth considering/discussing/ruling out whether this could possibly be a strain-specific pattern, i.e., does the identity of the competitor affect the way pre-insemination and post-insemination selection contribute to reproductive success?
   > We thank the reviewer for raising this point. We do not believe that the patterns are competitor strain-specific since experimental males were exposed to two different competitor strains throughout the experimental evolution and phenotypic assays. We discuss this point in the second paragraph of the discussion.

5. This is an interesting finding. If the genomic differences do not result in phenotypic differences, why exactly are they occurring? Could it be a difference in statistical power for phenotyping vs. genomic tests?
   > We agree with the reviewer that the difference between the phenotypic and genotypic results is interesting. Yes, we do believe that statistical power is likely the reason and have noted this in the discussion.

6. Did you test whether the genetic manipulations you did to generate inducible male sterility have any effects on mating success per se? If so, and you ruled it out, it would be good it mention it. If not, it may be good to add a caveat to that affect.
   > In the original sterility induction paper (Kasimatis et al. 2018 G3) we test the effect of the degron tag on fecundity. While it has does slightly decrease fecundity, this effect should not change the interpretation of our results as all experimentally evolving have this genetic manipulation. We have added this caveat to the methods section.

7. Not clear what ‘future stocks’ means.
   > Clarified (frozen for future research project).

8. A trivial point, but the octopus-like cartoons in the figure under “sperm dynamics” are somewhat jarring to me.
   > We agree the cartoons are unusual, however, they best represent the amoeboid-like shape of activated nematode sperm.

Reviewer #2

"Post-insemination selection dominates pre-insemination selection in driving rapid evolution of male competitive ability" utilizes the advantages of *C. elegans* to uniquely parse apart sexual selection on pre- and post-insemination. The experimental design is fully factorial to test total or post-insemination sexual selection when competition occurs either within or between populations. After 10 selection cycles spread across 30 generations the authors show differences in competitive insemination success against a novel competitor and sequencing of the different populations indicates that the this is polygenic with many peaks associated with this trait. Utilizing the genetic tools in *C. elegans* appears to be a very creative approach to tease apartment these questions, but I remain stuck on an aspect of the experimental design that makes it difficult for me to interpret the subsequent results.

> We appreciate the reviewer's careful critique and identification of areas of the design that were not fully explained. We have substantially revised and expanded our description of the design and partitioning of selection to address these comments, as well as adding a supplemental figure that aligns the experimental timeline with *C. elegans* development. Below we address each concern in detail.

Major Concerns:

The authors compare total (pre + post insemination selection) to only post-insemination selection. From my understanding, the pre-insemination competition is eliminated by sterilizing the worms using an Auxin induced system to stop sperm production. This experiment occurs on Day 1, so does it mean that females are only inseminated with sperm from worms that are the first to copulate, before the auxin begins to take effect? If this is the case, it seems like there is still pre-insemination competition and there is selection for fastest to copulate. I didn't see evidence for this, but wondered if perhaps the SPE-44 degron at an intermediate frequency in the population so only some of the worms are sterilized? The graphical images (figure 1, figure 2A) make it seem like the number of inseminated sperm decreases in this auxin treatment, but that does not seem congruent with the method described. It's possible that I'm missing something, but I have spent a lot of time rereading the methods and staring at the graphical cartoons and I can't fully understand what this step was selecting for, which makes it difficult to interpret if it is truly representing pre-insemination selection.

> We thank the reviewer for raising these points of clarification. We responded to each question separately.

> On insemination timing for the auxin treatments: In the original sterility induction paper (Kasimatis et al. 2018), we quantified that it takes 24hr for full sterility of males to be in effect (i.e., no more sperm produced and any stored sperm used). Males start producing sperm during larval stage 4. On day 1 of adulthood, the WS-PO and BS-PO replicates were transferred to auxin. This timing allowed for up to 24hr of mating between experimental males and females before full sterility was achieved. This timeline of events is shown in the new Figure S1. We agree with the reviewer that pre-insemination competition can (and likely did) occur within the experimental male population during this time. However, it is reduced relative to the non-auxin WS-P&P and BS-P&P regimes, which had 48hr of potential pre-insemination competition. We believe the first section of the results makes the differences between regimes clearer.

> On the frequency of *spe-44::degron*: The *spe-44::degron* locus is fixed in the experimental population (the locus was homozygosed prior to constructing the ancestral population) and the WS-PO and BS-PO replicates were screened every 3 generations during experimental evolution for correct function of the sterility locus, as stated in the methods.

> On the graphical representation: Figures 1 and 2 show a decrease in the number of green sperm (coming from experimental males) on day 2 of the experiment because after sterilization males cannot transfer more sperm and females are actively laying eggs during this time which uses sperm. We have clarified the Figure 1 legend and added Figure S1.

The introduction presents pre-insemination selection as mating success and it is unclear to me how sterilizing the worms reduces mating success. From what I understand about the system the worms may still "compete" for the female copulate and probably also copulate with them, just not successfully transfer sperm. My interpretation is that they are still reducing pre-insemination mating success while sterile, as they reduce the time non-sterile males may copulate and transfer their sperm.

> We appreciate the reviewer expanding on the subtlety of these effects. We address these in our response above and have modified the text to highlight the differences noted.

For the BS-PO experiment, does the temporal order of copulation effect the sperm usage? If the order of copulation plays a role in the sperm usage, it seems like this may not be selecting for post-insemination sperm success and instead just insemination order.

> We appreciate the reviewer raising this point of clarification. There is no evidence of temporal order of copulation effecting sperm usage in *C. elegans* (LaMunyon & Ward 1998). We have clarified this point in the results.

Please clarify if the ancestral males were pre- or post- EMS treatment. If the ancestral population has the genetic diversity introduced by the EMS and WS-P&P population is not undergoing selection, what is the hypothesis for the large difference in post-insemination success?

> We appreciate the reviewer raising these points of clarification. The ancestral population (G0) is post-mutagenesis (as stated in the first paragraph of the results and the methods). While direct selection was not applied to the WS-P&P regime, we cannot exclude that sexual selection happened "naturally" over the course of experimental evolution. We believe that the changes seen these regime replicates is a result of base-line or "natural" levels of sexual selection in worms. The re-written first paragraph of the results hopefully makes these distinctions clearer.

Is there a possibility that female choice is also evolving in these populations?

> We appreciate the reviewer raising this interesting point. We have collected separate RNA-seq data to address this question, which will be presented in a subsequent paper.

I think supplemental figure 4 that shows shared and unique peaks is interesting and should be considered including in the primary text.

> We agree with the reviewer and have moved this figure to the main text as Figure 4.

Does the sequencing of the G0s indicate if all of the significant SNPs exist in all of the founding populations, or is there the possibility of founders effect?

> We appreciate the reviewer's comment. Our genomic analyses required that a SNP be present in the ancestral population (G0) to be fit in both models 1 and 2. Therefore, all the significant SNPs were segregating in the ancestral population.

Minor Concerns:

1. Throughout the paper the authors refer to "evolving males"; this implies that there must be evolution occurring in this selection experiment. I would feel more comfortable if they were referred to as "experimental males", as this leaves it open to the results if all of the treatments resulted in evolution.
   > Changed as suggested.

2. Line 32-33: "we find that after 30 generations…" implies that selection occurred over all 30 generations, not the 10 selective rounds over 30 generations.
   > Corrected.

3. Line 43: not sure what is meant by "between the sexes". Please clarify.
   > Clarified (sexually dimorphic).

4. Line 183-186: "The BS-P&P and WS-PO regimes trended towards higher fraction of total reproductive success that could be attributed to post-insemination success across replicate means…" I think this references Figure 2C, but do not see this pattern in the data. Do you mean BS-P&Pand BS-PO? This trend is small, are there any statistics to back it up?
   > Yes, we are referencing BS-P&P and BS-PO in Figure 2C, which is now correctly stated. We apologize for the lack of clarity in our statement about the trend shown in Figure 2C. This effect is not significant and we have clarified this in the text. Please note that the lack of significance does not change any of our conclusions.

5. Have the significance levels in the fold-change comparisons (Figure 2D) been adjusted for multiple comparisons?
   > Yes, we used the default adjustment for multiple comparisons within the R package *multcomp*.

6. Adding the treatment as a title to each Manhattan plot in figure 3 would help comparing them easier.
   > Labels added.

7. Line 456: "The assay conditions mimicked the environment under which the worms evolved." I am unsure of what this means. My initial reading was each strain only received the treatments for which they had been selected (P&P vs PO and WS vs BS). But surely that can't be correct, as the within-strain experimental treatments that never experienced competition from another strain must have also been introduced to the novel competitor. Does it mean that only the WS-PO and BS-PO were exposed to the auxin before the competition experiment? If that is the case, are all the experimental worms sterile? Figure 2D suggests that all of the experimental conditions experienced both treatments.

> Clarified (the order and timing of sterility induction and addition of competition follows the experimental evolution timeline).

Reviewer #3
Kasimatis et al. apply a novel transgenic system to decouple pre- and post-copulatory competition in *C. elegans*. Factorial experimental evolution from induced mutations is followed by competitive fitness assays and pooled sequencing. The main findings are:
strong responses to selection across all four regimes, particularly in post-insemination competition
evolution was slowed by pre-insemination competition
the response to selection was polygenic, and most genes implicated are of unknown function
evolution of the X chromosome differed from autosomes

This work addresses an important question with a new, powerful method to isolate components of male fitness. Although large differences in response across regimes were not seen, the results obtained and utility of the method are noteworthy advances, in particular for our understanding of male *C. elegans* biology.

I have some minor issues with framing, and some technical issues that need to be addressed (which could possibly change the results a little, but not much I think), detailed below. Other questions/suggestions are commented in the pdf.

> We appreciate the reviewer's comments and attention to the genomic methods. We have redone or corrected analyses as the reviewer suggested. Below we respond to each concern in detail.

Framing:
In general, the paper is written with a lot of worm biology assumed, such as reproductive schedules. Please try and flesh this out in the introduction and methods to make the paper more accessible to others. For example, when is sperm production happening in males, at the relevant points during experimental evolution?

> We appreciate the reviewer raising this point of clarification. We have expanded the first section of the results to clarify the experimental design. In addition, Figure S1 addresses how the experimental timeline corresponds with spermatogenesis and female reproductive status.

The suggestion that post-insemination dynamics are neglected seems a bit overdone, given the enormous body of literature on the importance of gametic interactions (e.g. in free-spawning animals). Similarly, for organisms without flashy phenotypes (e.g. worms), there is of course ample precedent for the predominance of evolution in "the unseen world of molecular interactions".

> We appreciate the reviewer's comments and agree that substantial work has been done on gametic selection, especially in broadcast spawning organisms. We believe these studies are important and have added references in them in the introduction. We have also rephrased our language in the introduction to make it clearer that post-insemination interactions per se are not understudied, but rather the interaction between selection occurring during pre- and post-insemination interactions.

Technical issues requiring attention:
In analysis of Ne, the union of called SNPs shared with the ancestor are used for each individual sample, which is therefore confounded with sequencing depth. It would be better to use a set of common markers for all estimates (r^2 for Ne~nSNPs is >0.25 at G31).

> We thank the reviewer for pointing out this confounding issue and have recalculated Ne using a common set of SNPs.

Secondly, the PlanII method of Waples is used, which has been shown to be upwardly biased due to neglecting additional sampling associated with poolseq data (Jonas et al, 2016). A quick reanalysis using some of the estimators from Jonas et al. (R package poolSeq) shows lower values (mean ~420-520 at G31 across the four regimes at a set of ~35k common [called in >63/73 samples] markers), and the X chromosome is similar to autosomes (still unexpected perhaps, but less so than a significantly larger Ne. Note that Jonas et al. also saw similar X/auto values in a reanalysis of some fly data).

> We thank the reviewer for highlighting the differences between estimators. When recalculating Ne using the common set of SNPs, we used the poolSeq package (as suggested) to calculate Ne using both the Jonas et al. and Waples methods. For thoroughness, we now present both estimators in text.

The authors have, reasonably, used only the terminal G31 sample for Ne estimation where drift variance is maximal. Looking all time points, at the chromosome level, using a set of common markers across all samples, there is some evidence for an effect of regime: Ne appears to have flatlined for the two between-strain competition, but not for within-strain competition regimes. Make of this what you will.

> We appreciate the reviewer taking the time to examine this extra effect. However, we continue to present Ne using only the terminal G31 samples as a best estimate.

>20% of the genome is repeat masked (proportionally much less for the X), but diversity stats do not seem to be adjusted for this.

> We thank the reviewer for catching that the SNP density estimates were not repeat masked. We have doubled checked all the ancestral diversity statistics and only repeat masked estimates are now presented in text.

There looks to be an error in the calculation for theta (line 123) - the window used is 10000, not 1000 bp.

> Thank you for catching this typo – it has been corrected. No qualitative statements or interpretations changed.

In analysis of the poolseq data, the Bonferroni cutoff is set as 1/(0.5*N), rather than alpha/N.

> Thank you for catching this typo – it has been corrected. No qualitative statements or interpretations changed.

I also ask in the pdf to compare results from a quasibinomial glm - binomial error can be unrealistic for poolseq data leading to a high false positive rate, though I suspect the difference will not be large for your analyses.

> We agree with the reviewer that a quasibinomial glm can be more accurate for pool-seq data. However, prior to submitting the manuscript we did run our models using this structure and found the quasibinomial to be hypersensitive to outlier samples. Therefore, we present the binomial models as a more stable estimate of selection.

Question:
Is anything happening on the mitochondrion?
> While we think this is an interesting question, we did not have enough mitochondrial SNPs (< 100) to make any conclusive statements.

Comments from pop-up notes:

1. Pre-fertilization? Some of these flashy phenotypes are around in aquatic animals.
   > Corrected.

2. Tautological. ‘winning’ is success enough.
   > Corrected.

3. The focus is on internal fertilization, but this introduction could benefit, I think, from broadening to summarize some of the relevant findings from the enormous body of work on gamete interactions in free-spawning animals.
   > We appreciate the suggestion and have added references to gametic selection in broadcast spawning organisms.

4. Which populations?
   > Clarified (functional female-male populations).

5. And heat stress resistance.
   > Added.

6. This adjusted for repeat masking I assume?
   > Yes, diversity statistics were repeat masked.

7. It doesn’t look all that even by eye, add a summary statistic to compare with theta below? How does it compare to the distribution expected from base composition and the EMS mutation spectrum?
   > We thank the reviewer for this suggestion and have added summary statistics for the arm-center comparison of SFS. We believe that trying to estimate the EMS mutation spectrum given our mutagenesis methods would be highly inaccurate and therefore not a supportive addition to the results.

8. Again, worth specifying that this is only the terminal G30 evolved population. The intermediates are ‘evolved’, too.
   > Corrected.

9. No need to guess – are the proportions of SNPs/peak as expected from ancestral mutation density?

> We thank the reviewer for their suggestion. We have added a chi-squared statistic and changed our language to reflect the over-representation of peaks on chromosome arms.

10. Do these overlap with known hyper-divergent regions?
    > We appreciate the reviewer raising this interesting question. Approximately 79% of significance peaks overlap with hyper-divergent regions. We have added this information to the results section and File S6.

11. Except the ratios are different and density-dependent effects cannot be ruled out.
    > We have clarified our language about the phenotypic assays. The timing for sterility induction and addition of competitors follows the experimental evolution timeline.

12. For each regime x replicate (G > 0)?
    > Clarified.

13. Diallelic please! Apparently, the French are to blame for mixing Latin and Greek, despite precedence for the non-chimeric form.
    > Corrected.

14. This appears to link to a script that masks N sequences in a fasta file, not repeat regions? (unless the input is repeat masked with Ns which is not noted and would be unusual)
    > We have clarified our repeat making method. The code linked to in the methods generates a file of masked ranges using the *masked* N2 genome as a reference. These ranges were then filtered out of our dataset using R. We have clarified our process in the methods section.

15. Why fewer than above? You're considering variants that were called only in derived samples?
    > Thank you for catching this typo – the larger number was before repeat masking. We have corrected the total number of SNPs to be 321,929, which is the total after all QC and repeat masking.

16. Dispersion is likely to be very different from 1 due to the various sampling and pooling steps and so a quasibinomial error distribution is much more appropriate for pool-seq data (see Wiberg et al. 2017). Also, given your coverage is quite variable across time (and X/autosomes and presumably across loci) you should consider bias-correcting your counts (see e.g., Feder et al. 2012).
    > We appreciate the reviewer's suggestions. As mentioned above, we found the models fit with a quasibinomial error distribution to be more sensitive to outlier samples than those fit with a binomial error distribution. While we agree with the reviewer that variation in coverage can lead to mis-identification of significant SNPs, we believe our downstream analyses both in modeling fitting and calling significance peaks are conservative with respect to this concern.

17. There are size replicates per regime? I guess you mean samples (regimes x replicates x generation)?
    > Clarified (samples: regime x replicate).

18. You’re only interested in parallel evolution, but it still might be worth fitting replicate if you have heterogeneity (due to depth, worm sampling, etc).
> We thank the reviewer for this suggestion, but believe we have the most power and confidence in estimating selection as done.

19. Why five? And are the results sensitive to this choice?
> We appreciate the reviewer for raising this point. We selected five SNPs for defining peaks so as to capture the full linkage disequilibrium structure caused by a region under selection. Five SNPs is a conservative estimate of significance peaks. We re-did the analysis using 3 SNPs to define peaks and see slight differences, though overall the patterns are similar. We present both estimators in text and in File S6. We are reluctant to use less than three SNPs as we want to capture peaks caused by selection and not allele frequency change due to drift (which won’t necessarily have the same LD pattern).
